# Self-report psychopathy-III facet scores predict sexual crimes, sexual preferences, and sexual deviance index validity more precisely than total scores

**DOI:** 10.3389/fpsyg.2024.1359720

**Published:** 2024-03-25

**Authors:** Shaina A. Gabriel, Patrice Renaud

**Affiliations:** ^1^Department of Psychoeducation and Psychology, University of Quebec in Outaouais, Gatineau, QC, Canada; ^2^Forensic Immersion Laboratory, Philippe-Pinel National Institute of Legal Psychiatry, Montreal, QC, Canada; ^3^Centre for Research and Innovation in Cybersecurity and Society (CRICS), University of Quebec in Outaouais, Gatineau, QC, Canada

**Keywords:** psychopathy, self-report psychopathy-III, sexual crimes, sexual preference, sexual deviance index validity

## Abstract

Understanding the profiles of sexual offenders, such as the presence of psychopathic traits, is key to preventing future sexual crimes. The self-report psychopathy-III (SRP-III) is a tool used to assess the characteristics of psychopathy, but improvements on its interpretation are required to maximize its precision. The SRP-III can be interpreted by examining the scores on each of the four facets (interpersonal manipulation, callous affect, erratic lifestyle, antisocial behavior), on each of two factors (factor 1, factor 2), or by examining the total score. Here, we investigate the interpretation of the results from the SRP-III using these three approaches of analysis of the data for predicting types of sexual crimes, sexually deviant preferences (measured via PPG), and the validity of the sexual deviance results. Logistic regressions were carried out using either the four facets, two factors, or the total score of the SRP-III. Data were previously obtained from 198 Canadian men who were convicted of, or who admitted to committing, at least one sexual crime, or who reported experiencing sexually deviant fantasies. We also examined the point-biserial correlations between each of the methods of interpreting the SRP-III results and each of the dependent variables. We find that SRP-III facet scores most precisely predict types of sexual crimes, sexually deviant preferences, and sexual deviance index validity, followed by SRP-III factor scores, and lastly SRP-III total scores. Additionally, significant correlations are only found between SRP-III scores and one dependent variable. Potential reasons for this are discussed. Based on these findings, we recommend that future studies consider facet and factor scores in addition to the standard practice of examining total scores.

## 1 Introduction

According to self-report measures from 2019, there were 30 incidents of sexual assault per 1,000 Canadians in the age category of 15 years and above (Statistics Canada, 2021). This signifies an increase from 2014, when there were 22 incidents of sexual assaults per 1,000 Canadians aged 15 years and above (Conroy and Cotter, 2017). Such findings emphasize the need for tools to reduce the occurrence of sexual assaults, including the occurrence of sexual recidivism. Although a recent meta-analysis has found that the occurrence of sexual recidivism in Canada has declined over the past 80 years, it still occurs in approximately 10% of offenders (Lussier et al., 2023). Of importance to continuing this decline is knowledge on sexually deviant preferences, as they have been demonstrated to predict recidivism in sex offenders (Hanson and Bussière, 1998; Hanson and Morton-Bourgon, 2005; Olver and Wong, 2006; McPhail et al., 2019). This information can guide the decision-making process of correctional services when granting parole to those convicted of sexual crimes (Plaud, 2019). According to the DSM-5, a sexual preference is deemed to be deviant (or *paraphilic*) when there is an intense and persistent sexual preference for anything other than genital stimulation or preparatory fondling with a physically mature, consenting, and phenotypically normal human (American Psychiatric Association, 2013).

Sexually deviant preferences are generally measured using a penile plethysmography (PPG), the gold standard for measuring sexual arousal in men (Murphy et al., 2015), which was initially developed in 1957 by Freund (1963). PPG is a device that measures differences in penile circumference, which is used as an indicator of the level of arousal to sexually deviant and non-deviant stimuli. This can be used to distinguish a non-deviant sexual preference from a deviant sexual preference (Murphy et al., 2015). It has been shown that, although it offers part of the explanation, sexually deviant preferences do not entirely predict sexual crimes (Laws, 2009). Additional insights can be gained when the level of psychopathy is also considered (Hawes et al., 2013; Seto et al., 2016).

Psychopathy is a construct defined by Robert Hare as a combination of affective, interpersonal, and behavioral characteristics, which include egocentricity, impulsivity, irresponsibility, superficial affect, a lack of empathy, guilt or remorse, pathological lying, manipulation, and persistent violation of societal norms (Hare, 1996). Studies have linked higher levels of psychopathy to sexual recidivism (Looman et al., 2005; Parent et al., 2011). Studies have also shown that the combination of sexually deviant preferences and high psychopathy scores predict higher levels of sexual recidivism in a shorter period (Serin et al., 2001; Hildebrand et al., 2004; Hanson and Morton-Bourgon, 2005; Olver and Wong, 2006). However, there are certain inconsistencies in relation to these findings. For instance, some have found that psychopathy is linked to general offenses (i.e., all types), but not sexual offenses (Brown and Forth, 1997; Porter et al., 2009). Additionally, others have found that psychopathy is linked to general recidivism, but not sexual recidivism (Dietrich et al., 2007; Rettenberger et al., 2010; Harris et al., 2017; Yoon et al., 2022). These results suggest that our knowledge is incomplete and there is more to understand about the link between psychopathy, sexual offenses, and sexual recidivism.

The Psychopathy Checklist-Revised (PCL-R, Hare, 2003) is the gold standard for the measurement of psychopathy. The PCL-R consists of 20 items, measured on a three-point scale from 0 to 2 (where 0 = not present, 1 = partially present, and 2 = fully present), which make up two factors and four facets. Factor 1 contains the “interpersonal” and “affective” facets, which measure traits such as lack of empathy and remorse, and the presence of grandiosity and manipulation. Factor 2 contains the “lifestyle” and “antisocial” facets, which measure social deviance and an impulsive, irresponsible, and parasitic lifestyle. The total score is generally used to identify psychopathy, with cutoff scores of 25 (Harris et al., 2015) and 30 (Hare, 2006) out of 40 on the PCL-R indicating the occurrence of psychopathy.

A suboptimal interpretation of PCL-R scores may be contributing to discrepancies among studies investigating the link between psychopathy and sexual crimes. Certain authors have suggested that additional insights can be gained by investigating the link between sexual offenses and the factors and facets of the PCL-R (Brown et al., 2015; Krstic et al., 2018). For example, Hawes et al. (2013) have found that the total score, factor 2, and the antisocial facet predict sexual recidivism. However, the studies linking factors and facets of the PCL-R to sexual crimes remain sparse.

Given that the PCL-R is administered in an interview format requiring a trained interviewer, self-report measures have been developed for practical reasons. For example, the Self-Report Psychopathy-III (SRP-III) is a questionnaire that measures psychopathy following the same factor structure as the PCL-R (Paulhus et al., 2009). Self-report measures of psychopathy have been found to be negatively correlated to positive impression management bias (Sellbom et al., 2018), indicating that participants are unlikely to distort their answers to appear positively. Additionally, like the PCL-R, the SRP-III is able to distinguish those high on psychopathic propensities from those who are not (Neumann et al., 2015). The psychometric properties demonstrating the validity and reliability of the SRP-III are described in the materials section below.

When using the PCL-R and the SRP-III, the total score of psychopathy is generally used rather than separately evaluating individual factor or facet scores. However, reporting a high psychopathy total score does not provide information on how respondents scored on individual factors and facets, which could allow for a more precise profiling of sex offenders. Here, in line with our hypotheses, we investigate whether the SRP-III facet scores more precisely predict sexual crimes, sexual preferences, and sexual deviance index validity (an indicator of whether the evaluation of sexual preference was valid or not) than SRP-III factor scores, and whether SRP-III factor scores are a better predictor of these than SRP-III total scores. This could allow us to optimize our use of the SRP-III to enhance our understandings of the psychopathic profile of sex offenders, which may improve precision in predicting sexual recidivism.

## 2 Method

Secondary data that were previously archived are used in this study. Data were accessed according to the procedure in place at the Philippe-Pinel National Institute of Forensic Psychiatry (IPPM). Institutional review board approval was obtained from the research ethics committee of the University of Quebec in Outaouais.

### 2.1 Participants

Participants were 198 Canadian men that were convicted of, or who admitted to committing, at least one sexual crime, or who reported experiencing sexually deviant fantasies. The number of participants per category is listed in Table 1. The average age of the sample is 36.6 (SD = 14.2) years, with a range of 17–77 years. Participants were either sent by the court, by an outpatient clinic, or were patients interned at IPPM, a maximum-security psychiatric institution. The percentage of participants from each referral source and other demographic information (such as the ethnicity, income level, and IQ) are not reported as the authors do not have access to this information. The evaluations took place at the Forensic Immersion Laboratory of IPPM, which is one of the laboratories responsible for the assessments of individuals convicted of a sexual crime in the province of Quebec (Morissette, 2000). The initial sample comprised 368 participants, however all those who did not complete the SRP-III in full were excluded from the analyses. Reasons included refusal to fill out the questionnaire, accidentally missing items, or the questionnaire was filled out but not added to the dataset.

**Table 1:** Number of participants per category.

Categories	Number of participants
Crime committed against a minor with contact^a^	71
Crime committed against a minor without contact^a^	51
Crime committed against an adult with contact^a^	21
Crime committed against an adult without contact^a^	7
Crime committed against an adult and a minor with contact^b^	5
Crime committed against an adult and a minor without contact^b^	3
Self-reported pedophilic fantasies^c^	7
Self-reported crime against a minor with contact^c^	6
Crime committed as a minor (evaluated as an adult)^c^	21
Category unknown^c^	6

^a^Data from these participants were used in all 18 logistic regressions.

^b^Data from these participants were used in all logistic regressions except for those with the dependent variable “crime committed against an adult or a minor” (15 logistic regressions).

^c^Data from these participants were only used in the logistic regressions with dependent variables related to sexual preference and sexual deviance index validity (12 logistic regressions).

### 2.2 Materials

#### 2.2.1 Sociodemographic information

The type of sexual crime committed was obtained from each participant’s referral source. Additionally, an interview-format questionnaire was administered to all participants to obtain their sociodemographic information, including their age, and other information that was not used for the current study, such as their level of education, marital status, and source of revenue.

#### 2.2.2 Self-report psychopathy-III

The French version of the *Self-Report Psychopathy-III*, which was developed and validated by Gagnon (2011), was used in order to assess the presence of psychopathic traits. The SRP-III is a 64-item self-report questionnaire that measures psychopathy (Paulhus et al., 2009). The responses are measured on a five-point Likert scale, ranging from 1 (“Strongly disagree”) to 5 (“Strongly agree”). As previously mentioned, this tool follows the factor and facet structure of the PCL-R (Hare, 2003). The SRP-III was used rather than the PCL-R for pragmatic reasons, as no evaluator was qualified to administer the PCL-R, which is typically administered in interview format.

Factor 1 measures the Interpersonal manipulation facet (containing 16 items such as “I think I could beat a lie detector”) and the Callous affect facet (containing 16 items such as “I like to see fistfights”). Factor 2 measures the Erratic lifestyle facet (containing 16 items such as “I’ve often done something dangerous just for the thrill of it”) and the Antisocial behavior facet (containing 16 items such as “I have tricked someone into giving me money”). The SRP-III can be interpreted by summing the total score, the scores on each of the two factors, or the score of each of the four facets (Hare, 2006). The construct validity has been shown to be satisfactory (Williams et al., 2003) and the internal consistency (Cronbach’s α) of the total score is excellent (*α* = 0.94) and is acceptable for the subscales (0.74 ≤ α ≤ 0.86) (Sandvik et al., 2012). The bivariate test–retest reliability is excellent for the total score (*r* = 0.92) and is acceptable for the subscales (0.70 ≤ *r* ≤ 0.92) (Gordts et al., 2017).

#### 2.2.3 Penile plethysmography

PPG was used to measure the erectile response to various stimuli to determine the participants’ sexual preferences. The level of arousal is measured using a mercury-in-rubber strain gage manufactured by Limestone Technologies placed on the shaft of the penis, which measures changes in circumference. This is one of the most well validated measures of sexual response in men for research purposes (Murphy et al., 2015). Its discriminant validity is also well documented (Kalmus and Beech, 2005).

### 2.3 Procedure

First, an interview was conducted to collect participants’ sociodemographic information. Next, the SRP-III was filled out. Finally, sexual preference was measured using PPG. Participants were seated in an immersive vault composed of four white walls on which stimuli could be projected. They were instructed to place the mercury-in-rubber strain gage around the shaft of their penis and to wear headphones, as some of the stimuli involved sound. Additionally, an EEG helmet and glasses to allow for eye-tracking were used, however this data was not analyzed in the current study. The first measure was a control, where participants watched an audiovisual pornography, which acted as an indicator of the maximal penile response. In order to account for differences in sexual interest, the type of pornography was selected by the technician based on the nature of the sexual crime committed and the information collected during the interview.

Next, audio recordings depicting the following scenarios were played: neutral scenarios, heterosexual consensual relations with an adult, rape of a woman centered on humiliation, rape of a woman centered on physical violence, non-sexual physical assault of a woman (these scenarios were developed by Quinsey and Chaplin (1988) and were translated to French and validated by Barsetti et al. (1998)), incestuous sexual relations, sexual assault of a child without coercion, sexual assault of a child with coercion, and rape of a child with excessive violence (these scenarios were developed by Abel et al. (1978) and were translated to French and validated by Earls and Proulx (1986) and Proulx et al. (1994)). Twenty-six recordings were used for participants having sexually offended against a minor, whereas 13 recordings were used for those having sexually offended against an adult. Participants were attributed a sexual deviance score which classified them as “non deviant,” “possibly deviant,” or “deviant” based on whether they were more aroused by the consensual or nonconsensual scenarios.

Afterwards, visual stimuli were presented to participants. These were virtual synthetic characters of both sexes that were aged 6–7 years old, 10–11 years old, or 25 years old. The characters were programmed to make small nonsexual movements and showed neutral emotion. Two series of random character presentation orders were used. This made it possible to verify that the order in which the characters were shown did not influence the results. Each stimulus was presented for one and a half minutes, after which there was a 30 s break. This was extended if the penile response had not yet returned to baseline. Participants were attributed a sexual deviance score which classified them as “non deviant,” “possibly deviant,” or “deviant” based on whether they were more aroused by the adult or child characters.

A sexual deviance score for the auditive stimuli was calculated by dividing the maximal penile response to the nonconsensual scenarios by the maximal penile response to the consensual scenarios. Similarly, a sexual deviance score for the visual stimuli was calculated by dividing the maximal penile response to the child characters by the maximal penile response to the adult characters. A deviance index score between 0 and 0.79 indicates a non deviant profile, a deviance index score between 0.80 and 1.19 is indicative of a possibly deviant profile, and a deviance index score of 1.20 or higher indicates a deviant profile (Michaud and Proulx, 2009). However, if none of the stimuli provoked a change of circumference of at least 3 mm, the results were declared invalid (Michaud and Proulx, 2009).

### 2.4 Variables and statistical analyses

The independent variables are: (i) the total score on the SRP-III, (ii) the two factor scores of the SRP-III, and (iii) the four facet scores of the SRP-III. The dependent variables are (i) sexual offense with or without victim contact, (ii) sexual offense of at least one minor or at least one adult (this does not include mixed offenders), (iii) valid or invalid sexual deviance index for the auditive stimuli (consensual or nonconsensual sexual scenarios), (iv) valid or invalid sexual deviance index for the visual stimuli (adults or children), (v) preference for consensual sexual scenarios (non deviant sexual preference), nonconsensual sexual scenarios (deviant sexual preference), or between both (possibly deviant sexual preference), and (vi) sexual preference for adults (non deviant sexual preference), children (deviant sexual preference), or between both (possibly deviant sexual preference).

Since the independent variables are continuous and the dependent variables are nominal, binomial and multinomial logistic regressions were conducted using the software IBM SPSS Statistics for Windows (RRID:SCR_016479) version 29.0. A total of 18 logistic regressions were conducted linking the SRP-III total score, both SRP-III factor scores, or all four SRP-III facet scores with each dependent variable. For example, to test the dependent variable “crime with or without victim contact,” one model was constructed using the total scores, one model used both factor scores, and one model used all four facet scores. Then, Nagelkerke’s *R^2^* (an indicator of goodness-of-fit) were compared to determine which independent variable better predicts each of the dependent variables. Nagelkerke’s *R*^2^ is an adjustment of the Cox and Snell *R*^2^ which allows for the value to be situated between 0 and 1 (Field, 2018). A higher *R*^2^ indicates a better prediction of the dependent variable. In order to test the assumption of multicollinearity, the Pearson correlations between the two factors and between the four facets were verified. A Pearson correlation of *r* ≥ 0.90 was used to indicate the presence of multicollinearity.

Subsequently, point-biserial correlations were conducted using SPSS in order to examine the link between each facet, factor, and total score of the SRP-III and the type of sexual crime, sexual preference, and sexual deviance index validity. This resulted in 42 correlations, necessitating a Bonferroni correction of the *p*-value to *p* < 0.001. The assumptions of these correlations were verified. Normality was assessed based on whether the asymmetry and kurtosis values were between −2 and + 2, and homogeneity of variance was tested using Levene’s test of homoscedasticity.

## 3 Results

Table 2 contains Nagelkerke’s *R^2^* of 18 logistic regressions, linking each of the 3 independent variables to each of the 6 dependent variables. Results indicate that Nagelkerke’s *R*^2^ is higher for the facet scores than for the factor scores on all six dependent variables. For example, for the variable of crime committed with or without victim contact, Nagelkerke’s *R*^2^ = 0.081 for the SRP-III facet scores and Nagelkerke’s *R*^2^ = 0.033 for the SRP-III factor scores. Additionally, Nagelkerke’s *R*^2^ is higher for the factor scores than for the total scores on all six dependent variables. For instance, for the variable of crime committed with or without victim contact, Nagelkerke’s *R*^2^ = 0.024 for the SRP-III total score. Overall, the SRP-III facet scores predict sexual crimes, sexual preference, and sexual deviance index validity more precisely than the SRP-III factor scores, and SRP-III factor scores predict sexual crimes, sexual preference, and sexual deviance index validity more precisely than SRP-III total scores. Data from the logistic regressions can be found in Supplementary Tables S1–S18. Supplementary Table S19 summarizes the SRP-III scores of the sample. Supplementary Table S20 shows the sample size (*n*) per category for each dependent variable. This differs from Table 1 as it accounts for missing data for each variable. Supplementary Table S21 contains the Pearson correlations that allowed for the verification of the postulate of multicollinearity. This assumption was met for all independent variables.

**Table 2:** Nagelkerke’s *R*^2^ obtained from logistic regressions linking SRP-III total scores, SRP-III factor scores, and SRP-III facet scores to the type of sexual crime, sexual preference, and sexual deviance index validity.

Type of sexual crime, sexual preference, or sexual deviance index validity	SRP-III Total score	SRP-III Factor scores	SRP-III Facet scores
Crime with or without victim contact	0.024	0.033	0.081
Crime committed against an adult or a minor	0.172	0.229	0.237
Valid or invalid audio sexual deviance index	0.090	0.092	0.105
Valid or invalid visual sexual deviance index	0.099	0.102	0.136
Sexual preference for consensual or nonconsensual relations	0.058	0.078	0.114
Sexual preference for adults or children	0.062	0.142	0.380

Table 3 indicates the Pearson correlation between each SRP-III facet score, factor score, and the total score, and all six dependent variables. The only variable with significant correlations is “crime committed against an adult or a minor,” with the total score, Factor 2, Erratic lifestyle facet, and Antisocial behavior facet being significantly negatively correlated to having committed a crime against a minor. This indicates that a higher score is linked to a greater likelihood of offending against an adult rather than a minor. However, it is important to note that the only correlation among these that meets the assumption of homoscedasticity is the Erratic lifestyle facet. Postulates for the correlations are reported in Supplementary Tables S22, S23. It is also worth noting that the correlation between the Antisocial behavior facet and the variable “crime committed with or without contact” approaches significance (*p* = 0.004). It is possible that it did not reach significance due to the data for this correlation not satisfying the assumption of homogeneity of variance.

**Table 3:** Point-biserial correlations linking SRP-III total scores, SRP-III factor scores, and SRP-III facet scores to the type of sexual crime, sexual preference, and sexual deviance index validity.

Type of sexual crime, sexual preference, or sexual deviance index validity		SRP-III Total score	SRP-III Factor 1 score	SRP-III Factor 2 score	SRP-III Interper-sonal mani-pulation facet score	SRP-III Callous affect facet score	SRP-III Erratic lifestyle facet score	SRP-III Anti-social beha-viour facet score
Crime with or without victim contact	Pearson correlation	0.132	0.078	0.153	0.092	0.044	0.068	0.228
	Sig.	0.099	0.333	0.056	0.251	0.582	0.396	0.004
Crime committed against an adult or a minor	Pearson correlation	−0.341^*^	−0.205	−0.392^*^	−0.193	−0.169	−0.318^*^	−0.380^*^
	Sig.	< 0.001	0.012	< 0.001	0.018	0.039	< 0.001	< 0.001
Valid or invalid audio sexual deviance index	Pearson correlation	−0.247	−0.236	−0.213	−0.205	−0.225	−0.238	−0.110
	Sig.	0.013	0.018	0.033	0.041	0.024	0.017	0.278
Valid or invalid visual sexual deviance index	Pearson correlation	−0.269	−0.227	−0.268	−0.164	−0.253	−0.288	−0.155
	Sig.	0.017	0.044	0.017	0.148	0.025	0.010	0.173
Sexual preference for consensual or nonconsensual relations	Pearson correlation	0.100	0.065	0.118	0.040	0.083	0.059	0.155
	Sig.	0.426	0.603	0.346	0.751	0.509	0.638	0.213
Sexual preference for adults or children	Pearson correlation	0.019	0.039	−0.004	0.116	−0.058	−0.185	0.190
	Sig.	0.908	0.810	0.979	0.477	0.723	0.252	0.240

*Correlation significant with *p* < 0.001 (following a Bonferroni correction).

## 4 Discussion

Psychopathy scores were interpreted using the SRP-III total score, the SRP-III factor scores, and the SRP-III facet scores in order to evaluate which approach most precisely predicts sexual crimes, sexual preferences, and sexual deviance index validity. Nagelkerke’s *R*^2^ was used as an indicator of goodness-of-fit. In accordance with our hypotheses, SRP-III facet scores were consistently found to be the most precise in predicting sexual crimes, sexual preferences, and sexual deviance index validity. Additionally, the SRP-III factor scores were more precise than using the SRP-III total scores and they were less precise than the SRP-III facet scores.

The higher precision observed from using SRP-III facet scores may be due to the consideration of the scores on all four SRP-III facets, which provides information on the amount of variance that can be explained by each score across four groups of characteristics (interpersonal manipulation, callous affect, erratic lifestyle, and antisocial behavior). For instance, as is reported in Table 3, the only correlation that approaches significance for the variable “crime committed with or without victim contact” is the antisocial behavior facet score (*r* = 0.228, *p* = 0.004). This is a case in which the score is more discriminant with the facet score than with the total score.

Similarly, SRP-III factor scores provide information on the amount of variance explained by each factor, which is lost when merely interpreting SRP-III total scores. For instance, as can be seen in Table 3, according to the correlations between the SRP-III scores and the variable “crime committed against an adult or a minor”, *r* is highest for the Factor 2 score (*r* = −0.392, *p* < 0.001), whereas *r* = −0.341 for the total score (*p* < 0.001). This illustrates the way that accounting for each factor or facet’s contribution to the prediction of sexual crimes, sexual preferences, and sexual deviance index validity allows for a higher degree of precision.

It is worth noting that the correlations linking the SRP-III scores to five out of the six dependant variables are non-significant. The null findings for the two sexual preference variables are expected due to the low sample sizes in each category (shown in Supplementary Table S20). Future studies using PPG and psychopathy measures could verify these findings using larger sample sizes. However, a correlation is expected between sexual deviance index validity and psychopathy scores due to response inhibition being negatively correlated with psychopathy (Gillespie et al., 2022). Despite the negative correlations noted in Table 3, these are not significant. This could be due to the Bonferroni correction increasing the probability of Type II error.

Given that the SRP-III follows the same factor structure as the PCL-R, these findings may also be applicable to PCL-R scores. For instance, in a study by Burt et al. (2016) comparing violently recidivating psychopathic offenders (RPO) to violent non-recidivating psychopathic offenders (non-RPO), it was found that although PCL-R total scores did not differ significantly, a significant difference was measured between the two groups when using the factor and facet scores. PCL-R scores from RPOs were higher on factor 2 and lower on factor 1, especially the interpersonal facet, than for the non-RPOs. Similarly, a study by Sohn et al. (2022) found that child sex offenders scored higher on the interpersonal and affective facets of the PCL-R than nonsexual offenders, while there were no significant differences in their total scores. Both of these studies provide instances where the analysis of the PCL-R facet and factor scores have allowed for two groups of offenders to be distinguished, while their PCL-R total scores were not sufficient to draw the same distinctions. These results are consistent with the findings from the SRP-III described above, indicating that facet scores and factor scores provide a more precise distinction of types of offenders than total scores alone.

Interestingly, in another study, Mokros et al. (2015) performed latent profile analysis on PCL-R and SRP-III scores to identify homogeneous subgroups using maximum likelihood estimation. Their results indicate that facet scores of male offenders can create psychopathic profiles consistent with clinical and empirical descriptions (Mokros et al., 2015). More specifically, a manipulative type characterized as passive, parasitic, and complex, an aggressive type, characterized as predatory and simple, and a sociopathic type, defined as individuals who were socialized to be antisocial in society and loyal to members of their own group (Mokros et al., 2015). Thus, these authors have demonstrated that the additional information provided by facet scores may allow us to characterize types of psychopathy, further supporting the utility interpreting facet scores.

Overall, the characterization of offenders using psychopathic profiles based on the SRP-III scores obtained on each of the facets and factors offers a higher degree of precision than simply using the SRP-III total scores. These findings suggest that future studies linking SRP-III scores to sexual crimes, sexual preferences, or sexual deviance index validity could improve the precision of the results by interpreting SRP-III facet and SRP-III factor scores in addition to the standard approach of interpreting SRP-III total scores. This could advance our understandings of the factors contributing to sexual crimes, including addressing certain inconsistences in the literature. Future studies could examine whether the SRP-III factor scores and SRP-III facet scores can be used in the same way when studying other types of crimes, such as non-sexual crimes. Additionally, future studies could investigate the applicability of these findings to the PCL-R and other psychopathy measures using the same structure.

Several limitations should be considered when interpreting these results. First, the exclusion criterion eliminated a substantial proportion of the sample. This may have introduced a selection bias in the participants. Out of the initial sample of 368 participants, those who did not complete all the items on the SRP-III comprised 46% of the sample. Therefore, the sample used for this study may not be representative of the target population (i.e., sexual offenders). However, we have noticed that trends expected based on the literature apply to our sample. For instance, there is a near significant correlation between higher antisocial behavior facet scores and participants who offended against adults rather than minors (*r* = 0.228, *p* = 0.004, Table 3), as has been previously reported (Sohn et al., 2022). Additionally, a link has previously been established between antisocial traits (as can be measured by factor 2 and the antisocial behavior facet of the SRP-III) and committing a crime with contact (Webb et al., 2007). This has been observed in our sample, as noted above. Second, even though the SRP-III has been validated, it has been criticized for having low correlations to the PCL-R interpersonal and affective facets (Sandvik et al., 2012; Ducro et al., 2016). Ideally, the current study would have used both SRP-III and PCL-R data, however PCL-R data was not available for the sample. Consequentially, it would be pertinent to test the predictive ability of factors and facets using other measures of psychopathy, including the PCL-R. However, as described above, we note that studies using the PCL-R have been able to gain insights when also considering factor and facet scores.

In conclusion, SRP-III facet scores were found to be the most precise in predicting sexual crimes, sexual preference, and sexual deviance index validity followed by SRP-III factor scores, and finally SRP-III total scores. Future studies linking the SRP-III, and potentially also the PCL-R, to these could benefit from interpreting the facet scores and factor scores rather than only using total scores. This method may also be helpful when studying other types of crimes, such as non-sexual crimes and could allow for the characterization of psychopathic profiles.

## Data availability statement

The data analyzed in this study is subject to the following licenses/restrictions: There are ethical contraindications to publishing the data set. Requests to access these datasets should be directed to patrice.renaud@uqo.ca.

## Ethics statement

This study involving humans was approved by the Director of Professional Services and Forensic Affairs of the Philippe-Pinel National Institute of Forensic Psychiatry and the Research Ethics Committee of the University of Quebec in Outaouais. The studies were conducted in accordance with the local legislation and institutional requirements. The ethics committee/institutional review board waived the requirement of written informed consent for participation from the participants or the participants’ legal guardians/next of kin because we are in compliance with the Act respecting Access to Documents held by Public Bodies and the Protection of Personal Information.

## Author contributions

SG: Conceptualization, Data curation, Formal analysis, Methodology, Project administration, Writing – original draft, Writing – review & editing. PR: Funding acquisition, Writing – original draft, Writing – review & editing.
