# Supplementary material for: Self-report psychopathy-III facet scores predict sexual crimes, sexual preferences, and sexual deviance index validity more precisely than total scores
Source: Front Psychol. 2024 Mar 25;15:1359720. doi: 10.3389/fpsyg.2024.1359720 (PMC10999618; doi:10.3389/fpsyg.2024.1359720)
Supplement: Supplementary file 1 [file Data_Sheet_1.PDF]

Supplementary Material

Self-Report Psychopathy-III Facet Scores Predict Sexual Crimes, Sexual Preferences, and Sexual Deviance Index Validity More Precisely Than Total Scores

Shaina A. Gabriel[1] & Patrice Renaud[1,2,3*]

[1]Department of Psychoeducation and Psychology, University of Quebec in Outaouais, Gatineau, Quebec, Canada

[2]Forensic Immersion Laboratory, Philippe-Pinel National Institute of Legal Psychiatry, Montreal, Quebec, Canada

[3]Centre for Research and Innovation in Cybersecurity and Society (CRICS), University of Quebec in Outaouais, Gatineau, Quebec, Canada

*** Correspondence:**
Patrice Renaud
patrice.renaud@uqo.ca

1 Supplementary Tables

Table S1 Results from the logistic regression predicting crime with or without victim contact using the SRP-III total scores.

	B[1]	Standard error	p-value	Odds ratio	Lower 95% confidence interval	Upper 95% confidence interval
Total score	.012	0.007	.101	1.012	0.998	1.026
Constant	-1.260	1.057	.233	0.284	-	-

[1]Beta is unstandardized.
* Reference (or baseline) category: without victim contact (no contact = 0; contact = 1)

Table S2 Results from the logistic regression predicting crime with or without victim contact using the two SRP-III factor scores.

	B[1]	Standard error	p-value	Odds ratio	Lower 95% confidence interval	Upper 95% confidence interval
Factor 1	-.005	0.018	.783	0.995	0.961	1.030
Factor 2	.025	0.015	.096	1.025	0.996	1.055
Constant	-1.069	1.068	.317	0.343	-	-

[1]Beta is unstandardized.

* Reference category: without victim contact (no contact = 0; contact = 1)

Table S3 Results from the logistic regression predicting crime with or without victim contact using the four SRP-III facet scores.

	B[1]	Standard error	p-value	Odds ratio	Lower 95% confidence interval	Upper 95% confidence interval
Interpersonal manipulation	.014	0.033	.672	1.014	0.951	1.081
Callous affect	-.020	0.032	.520	0.980	0.921	1.043
Erratic lifestyle	-.016	0.023	.489	0.985	0.942	1.029
Antisocial behaviour	.089	0.032	.006	1.093	1.026	1.164
Constant	-1.654	1.149	.150	0.191	-	-

[1]Beta is unstandardized.

* Reference category: without victim contact (no contact = 0; contact = 1)

Table S4 Results from the logistic regression predicting crime committed against an adult or a minor using the SRP-III total scores.

	B[1]	Standard error	p-value	Odds ratio	Lower 95% confidence interval	Upper 95% confidence interval
Total score	-.037	0.010	<.001	0.964	0.946	0.983
Constant	7.104	1.545	<.001	1217.146	-	-

[1]Beta is unstandardized.
* Reference category: crime committed against an adult (adult = 0; minor = 1)

Table S5 Results from the logistic regression predicting crime committed against an adult or a minor using the two SRP-III factor scores.

	B[1]	Standard error	p-value	Odds ratio	Lower 95% confidence interval	Upper 95% confidence interval
Factor 1	.013	0.022	.568	1.013	0.970	1.058
Factor 2	-.077	0.020	<.001	0.926	0.890	0.963
Constant	6.733	1.532	<.001	839.750	-	-

[1]Beta is unstandardized.
* Reference category: crime committed against an adult (adult = 0; minor = 1)

Table S6 Results from the logistic regression predicting committed against an adult or a minor using the four SRP-III facet scores.

	B[1]	Standard error	p-value	Odds ratio	Lower 95% confidence interval	Upper 95% confidence interval
Interpersonal manipulation	.023	0.043	.592	1.023	0.941	1.113
Callous affect	.004	0.041	.921	1.004	0.927	1.088
Erratic lifestyle	-.057	0.031	.065	0.945	0.890	1.003
Antisocial behaviour	-.108	0.040	.007	0.897	0.830	0.971
Constant	6.855	1.535	<.001	948.391	-	-

[1]Beta is unstandardized.
* Reference category: crime committed against an adult (adult = 0; minor = 1)

Table S7 Results from the logistic regression predicting audio sexual deviance index validity using the SRP-III total scores.

	B[1]	Standard error	p-value	Odds ratio	Lower 95% confidence interval	Upper 95% confidence interval
Total score	-.026	0.011	.017	0.974	0.953	0.995
Constant	3.158	1.590	.047	23.517	-	-

[1]Beta is unstandardized.
* Reference category: valid audio sexual deviance index validity (valid = 0; invalid = 1)

Table S8 Results from the logistic regression predicting audio sexual deviance index validity using the two SRP-III factor scores.

	B[1]	Standard error	p-value	Odds ratio	Lower 95% confidence interval	Upper 95% confidence interval
Factor 1	-.035	0.025	.166	0.966	0.919	1.015
Factor 2	-.019	0.023	.425	0.982	0.938	1.027
Constant	3.188	1.599	.046	24.238	-	-

[1]Beta is unstandardized.
* Reference category: valid audio sexual deviance index validity (valid = 0; invalid = 1)

Table S9 Results from the logistic regression predicting audio sexual deviance index validity using the four SRP-III facet scores.

	B[1]	Standard error	p-value	Odds ratio	Lower 95% confidence interval	Upper 95% confidence interval
Interpersonal manipulation	-.018	0.044	.686	0.982	0.901	1.071
Callous affect	-.052	0.048	.284	0.950	0.864	1.044
Erratic lifestyle	-.041	0.033	.216	0.959	0.899	1.025
Antisocial behaviour	.014	0.042	.742	1.014	0.933	1.102
Constant	3.011	1.630	.065	20.305	-	-

[1]Beta is unstandardized.
* Reference category: valid audio sexual deviance index validity (valid = 0; invalid = 1)

Table S10 Results from the logistic regression predicting visual sexual deviance index validity using the SRP-III total scores.

	B[1]	Standard error	p-value	Odds ratio	Lower 95% confidence interval	Upper 95% confidence interval
Total score	-.026	0.011	.023	0.974	0.952	0.996
Constant	3.801	1.690	.025	44.737	-	-

[1]Beta is unstandardized.
* Reference category: valid visual sexual deviance index validity (valid = 0; invalid = 1)

Table S11 Results from the logistic regression predicting visual sexual deviance index validity using the two SRP-III factor scores.

	B[1]	Standard error	p-value	Odds ratio	Lower 95% confidence interval	Upper 95% confidence interval
Factor 1	-.016	0.027	.552	0.984	0.933	1.037
Factor 2	-.036	0.026	.168	0.965	0.917	1.015
Constant	3.784	1.683	.025	43.989	-	-

[1]Beta is unstandardized.
* Reference category: valid visual sexual deviance index validity (valid = 0; invalid = 1)

Table S12 Results from the logistic regression predicting visual sexual deviance index validity using the four SRP-III facet scores.

	B[1]	Standard error	p-value	Odds ratio	Lower 95% confidence interval	Upper 95% confidence interval
Interpersonal manipulation	.028	0.048	.550	1.029	0.937	1.129
Callous affect	-.063	0.050	.213	0.939	0.851	1.037
Erratic lifestyle	-.066	0.038	.086	0.936	0.869	1.009
Antisocial behaviour	.001	0.045	.990	1.001	0.916	1.093
Constant	3.830	1.696	.024	46.083	-	-

[1]Beta is unstandardized.

* Reference category: valid visual sexual deviance index validity (valid = 0; invalid = 1)

Table S13 Results from the logistic regression predicting sexual preference for consensual or nonconsensual relations using the SRP-III total scores.

		B[1]	Standard error	p-value	Odds ratio	Lower 95% confidence interval	Upper 95% confidence interval
Non-deviant	Total score	-.016	0.015	.275	0.984	0.957	1.013
	Constant	1.857	2.131	.384	-	-	-
Poss. Deviant	Total score	.010	0.011	.370	1.010	0.988	1.033
	Constant	-1.787	1.790	.318	-	-	-

[1]Beta is unstandardized.

* Reference category: deviant sexual preference (deviant = 0; possibly deviant = 1; non-deviant = 2)

Table S14 Results from the logistic regression predicting sexual preference for consensual or nonconsensual relations using the two SRP-III factor scores.

		B[1]	Standard error	p-value	Odds ratio	Lower 95% confidence interval	Upper 95% confidence interval
Non-deviant	Factor 1	-.006	0.036	.865	0.994	0.927	1.066
	Factor 2	-.025	0.033	.447	0.975	0.913	1.041
	Constant	1.877	2.147	.382	-	-	-
Poss. Deviant	Factor 1	.041	0.030	.177	1.042	0.982	1.105
	Factor 2	-.020	0.029	.496	0.980	0.925	1.038
	Constant	-1.724	1.831	.347	-	-	-

[1]Beta is unstandardized.

* Reference category: deviant sexual preference (deviant = 0; possibly deviant = 1; non-deviant = 2)

Table S15 Results from the logistic regression predicting sexual preference for consensual or nonconsensual relations using the four SRP-III facet scores.

		B[1]	Standard error	p-value	Odds ratio	Lower 95% confidence interval	Upper 95% confidence interval
Non-deviant	Interpersonal manipulation	.011	0.056	.844	1.011	0.906	1.128
	Callous affect	-.027	0.064	.674	0.973	0.859	1.104
	Erratic lifestyle	.008	0.049	.866	1.008	0.917	1.109
	Antisocial behaviour	-.082	0.063	.196	0.921	0.814	1.043
	Constant	2.379	2.310	.303	-	-	-
Poss. Deviant	Interpersonal manipulation	.042	0.050	.405	1.042	0.945	1.149
	Callous affect	.042	0.060	.488	1.043	0.926	1.174
	Erratic lifestyle	-.036	0.046	.425	0.964	0.882	1.054
	Antisocial behaviour	-.001	0.052	.988	0.999	0.903	1.106
	Constant	-1.758	1.857	.344	-	-	-

[1]Beta is unstandardized.

* Reference category: deviant sexual preference (deviant = 0; possibly deviant = 1; non-deviant = 2)

Table S16 Results from the logistic regression predicting sexual preference for adults or children relations using the SRP-III total scores.

		B[1]	Standard error	p-value	Odds ratio	Lower 95% confidence interval	Upper 95% confidence interval
Non-deviant	Total score	-.003	0.015	.859	0.997	0.967	1.028
	Constant	.288	2.347	.902	-	-	-
Poss. Deviant	Total score	.022	0.017	.211	1.022	0.988	1.057
	Constant	-4.162	2.786	.135	-	-	-

[1]Beta is unstandardized.

* Reference category: deviant sexual preference (deviant = 0; possibly deviant = 1; non-deviant = 2)

Table S17 Results from the logistic regression predicting sexual preference for adults or children relations using the two SRP-III factor scores.

		B[1]	Standard error	p-value	Odds ratio	Lower 95% confidence interval	Upper 95% confidence interval
Non-deviant	Factor 1	-.018	0.044	.678	0.982	0.901	1.070
	Factor 2	.014	0.047	.760	1.014	0.925	1.112
	Constant	.131	2.311	.955	-	-	-
Poss. Deviant	Factor 1	-.059	0.051	.247	0.942	0.852	1.042
	Factor 2	.109	0.057	.054	1.115	0.998	1.246
	Constant	-5.025	2.892	.082	-	-	-

[1]Beta is unstandardized.

* Reference category: deviant sexual preference (deviant = 0; possibly deviant = 1; non-deviant = 2)

Table S18 Results from the logistic regression predicting sexual preference for adults or children relations using the four SRP-III facet scores.

		B[1]	Standard error	p-value	Odds ratio	Lower 95% confidence interval	Upper 95% confidence interval
Non-deviant	Interpersonal manipulation	-.086	0.072	.234	0.918	0.796	1.057
	Callous affect	.055	0.080	.490	1.057	0.904	1.235
	Erratic lifestyle	.143	0.078	.069	1.153	0.989	1.345
	Antisocial behaviour	-.118	0.076	.123	0.889	0.766	1.032
	Constant	-.973	2.553	.703	-	-	-
Poss. Deviant	Interpersonal manipulation	-.134	0.089	.133	0.875	0.735	1.042
	Callous affect	-.002	0.098	.987	0.998	0.824	1.210
	Erratic lifestyle	.320	0.120	.008	1.377	1.088	1.743
	Antisocial behaviour	-.054	0.087	.532	0.947	0.800	1.123
	Constant	-8.131	3.769	.031	-	-	-

[1]Beta is unstandardized.

* Reference category: deviant sexual preference (deviant = 0; possibly deviant = 1; non-deviant = 2)

Table S19 Descriptive statistics of the SRP-III total scores, SRP-III factor scores, and SRP-III facet scores from the sample (n = 198).

Type of SRP-III score	Maximum possible score	Average Score	Standard deviation	Minimum value	Maximum value
Interpersonal manipulation facet	80	37.00	7.35	24	67
Callous affect facet	80	35.69	6.68	20	61
Erratic lifestyle facet	80	42.60	9.34	17	64
Antisocial behaviour facet	80	34.57	7.06	24	55
Factor 1	160	72.69	12.54	46	125
Factor 2	160	77.18	14.37	46	119
Total score	320	149.86	24.21	101	240

Table S20 Sample size (n) per category for each dependent variable.

Type of sexual crime, sexual preference, or sexual deviance index validity	Sample size (n) category 1	Sample size (n) category 2	Sample size (n) category 3
Crime with or without victim contact	Without contact = 61	With contact = 97	–
Crime committed against an adult or a minor	Adult = 28	Minor = 122	–
Valid or invalid audio sexual deviance index	Valid = 66	Invalid = 34	–
Valid or invalid visual sexual deviance index	Valid = 40	Invalid = 39	–
Sexual preference for consensual or nonconsensual relations	Non-deviant = 17	Possibly deviant = 22	Deviant = 27
Sexual preference for adults or children	Non-deviant = 15	Possibly deviant = 8	Deviant = 17

Table S21 Pearson correlations between the two factors and the four facets to verify the postulate of multicollinearity of the logistic regressions.

**Type of SRP-III score**	Factor 1	Interpersonal Manipulation facet
Factor 2	0.617*	-
Callous Affect facet	-	0.597*
Erratic Lifestyle facet	-	0.558*
Antisocial Behaviour facet	-	0.461*

*p < .001

Table S22 Postulate of normality satisfied for each category for all six dependent variables (Y = Yes; N = No)

Type of sexual crime, sexual preference, or sexual deviance index validity	SRP-III Total score	SRP-III Factor 1 score	SRP-III Factor 2 score	SRP-III Interper-sonal mani-pulation facet score	SRP-III Callous affect facet score	SRP-III Erratic lifestyle facet score	SRP-III Anti-social beha-viour facet score
Crime with victim contact	Y	N[1]	Y	Y	Y	Y	Y
Crime without victim contact	Y	Y	Y	Y	Y	Y	Y
Crime committed against an adult	Y	Y	Y	Y	Y	Y	Y
Crime committed against a minor	Y	Y	Y	Y	Y	Y	Y
Valid audio sexual deviance index	Y	N[a]	Y	N[a]	Y	Y	Y
Invalid audio sexual deviance index	Y	Y	Y	Y	Y	Y	Y
Valid visual sexual deviance index	N[a]	N[a]	Y	N[a]	Y	Y	Y
Invalid visual sexual deviance index	Y	Y	Y	Y	Y	Y	Y
Sexual preference for consensual relations	Y	Y	Y	Y	Y	Y	Y
Sexual preference between both	Y	Y	Y	Y	Y	Y	Y
Sexual preference for nonconsensual relations	Y	Y	Y	Y	Y	Y	Y
Sexual preference for adults	Y	Y	N[1]	Y	Y	Y	Y
Sexual preference between both	N[a]	N[1b]	Y	N[b]	N[a]	Y	Y
Sexual preference for children	Y	Y	Y	Y	Y	Y	Y

1 = Asymmetry between -2 and +2; 2 = Asymmetry below -4 or above +4; a = Kurtosis between -2 and +2; b = Kurtosis below -4 or above +4

Table S23 Postulate of homogeneity of variances satisfied between the categories of all six dependent variables according to Levene's test of homogeneity (Y = Yes; N = No).

**Type of sexual crime, sexual preference, or sexual deviance index validity**	**SRP-III Total score**	**SRP-III Factor 1 score**	**SRP-III Factor 2 score**	**SRP-III Interper-sonal mani-pulation facet score**	**SRP-III Callous affect facet score**	**SRP-III Erratic lifestyle facet score**	**SRP-III Anti-social beha-viour facet score**
Crime with or without victim contact	Y	Y	Y	Y	Y	Y	N[a]
Crime committed against an adult or a minor	N[a]	N[a]	N[b]	N[a]	Y	Y	N[b]
Valid or invalid audio sexual deviance index	Y	Y	Y	Y	Y	Y	Y
Valid or invalid visual sexual deviance index	Y	Y	Y	Y	Y	Y	Y
Sexual preference for consensual or nonconsensual relations	Y	Y	Y	Y	Y	Y	Y
Sexual preference for adults or children	Y	Y	Y	Y	N[a]	Y	Y

a = $p < .05$; b = $p < .01$; c = $p < .001$
